# Double Trouble: The Perilous Intersection of Amoebic Colitis and Candida Infection Leading to Colonic Perforation

**DOI:** 10.7759/cureus.71292

**Published:** 2024-10-12

**Authors:** Mary Anelia Correya, Thanka Johnson, Shobana Balakrishnan, Rajendran S

**Affiliations:** 1 Department of Pathology, Sree Balaji Medical College and Hospital, Chennai, IND; 2 Department of Surgical Gastroenterology, Sree Balaji Medical College and Hospital, Chennai, IND

**Keywords:** amoeba, candida, colon, infection, perforation, ulcer

## Abstract

Amoebic colitis, a parasitic infection caused by *Entamoeba histolytica*, can lead to severe gastrointestinal symptoms. The clinical manifestations can vary widely, from being an asymptomatic carrier to experiencing severe colitis and even colonic perforation. Surgical treatment for fulminant amoebic colitis and colonic perforation should be carried out at the earliest. Superadded fungal infections in these patients are rare and can significantly complicate the clinical course. Here, we present a unique case of amoebic colitis complicated by a superadded Candida infection, culminating in colonic perforation.

## Introduction

Amoebic colitis is an infection of the colon caused by the protozoan parasite *Entamoeba histolytica*. Most individuals affected remain asymptomatic but some of the infected cases can develop severe gastrointestinal symptoms like abdominal pain and diarrhoea with discharge of mucous and blood. In certain severe cases, amoebic colitis can be further complicated by life-threatening colonic perforation. These severe cases of amoebic colitis that present with colonic perforation are rare and associated with a high mortality rate (>55%) [1]. The presentation of colonic perforation can be often compounded by secondary fungal infections. Colonization by fungal organisms like Candida, which are commonly found as opportunistic pathogens in immunocompromised or critically ill patients, can rarely cause superadded infections in necrotic colonic ulcers. This paper discusses the pathological mechanisms underlying amoebic colitis with colonic perforation and examines the role of Candida infections in exacerbating the condition.

## Case presentation

A 53-year-old, Asian Indian female came to the casualty with a sudden decrease in responsiveness. She had a history of abdominal pain in the left hypochondrium for a month, which was intermittent and non-radiating, and loose stools for 15 days with about 4-5 episodes per day. She also had a history of nausea, loss of appetite, and loss of weight for two weeks. She was a known case of type 2 diabetes mellitus, on treatment for the past 20 years. Personal and family histories were unremarkable in context to the present illness. On physical examination, she was normothermic and her vitals were within normal limits. An abdominal examination was done, which revealed tenderness over the left hypochondrium. There was no appreciable organomegaly.

Investigations

Complete blood count showed hemoglobin of 10 g/dl, total leukocyte count of 18.5x10^9/L, neutrophils of 86.1%, and platelet count of 244x10^3/L. The erythrocyte sedimentation rate value was 98 mm/hr. The C-reactive protein was 19.6mg/dl. Renal function tests, lipid profile, and electrolytes were within normal limits. The fasting blood glucose level was 200 mg/dl and glycated hemoglobin (HbA1c) was 7.44. Serological tests for HIV and hepatitis B surface antigen (HBsAg) were negative. The stool for occult blood was found to be positive. A contrast-enhanced CT scan revealed a large diverticulum with probable perforation and a heterogeneously enhancing collection adjacent to the splenic flexure of the colon; air foci were seen adjacent to the collection. A colonoscopy revealed multiple colonic ulcers with a large ulcer showing a diverticulum-like opening.

Differential diagnosis

A clinical differential diagnosis of splenic flexure perforation with a possibility of malignancy or an infective pathology was considered.

Treatment

The patient was taken for emergency laparotomy, and sub-total colectomy with end ileostomy was done and the specimen was sent for histopathological examination. 

Pathological examination

**Figure 1:**
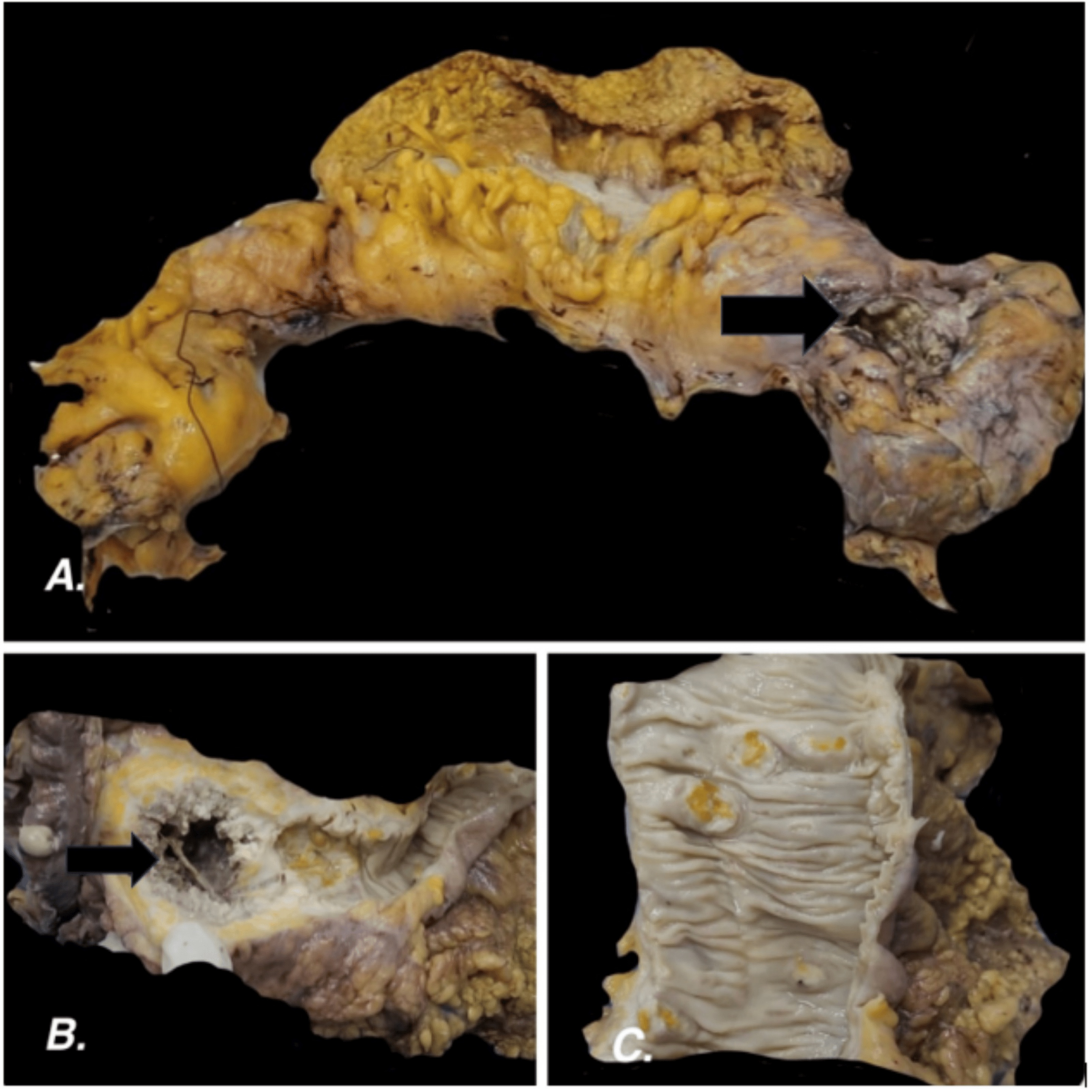
Gross examination 1A. Colectomy specimen showing perforation on the serosal aspect near the splenic flexure; 1B. The cut surface of the perforation area showing a necrotic ulcer with gray-white friable material; 1C. Other burrowing ulcers on the mucosal surface

On gross examination, the serosal surface showed a perforation measuring 3 cm in the greatest dimension (Figure 1A). The mucosal surface showed six burrowing ulcers. The largest ulcerative lesion was 6.9 cm in diameter and was filled with gray-white friable necrotic material with a wall thickness of 2.3 cm (Figures 1B, 1C). Focal areas of mucosal flattening were noted. The specimen was gross-examined and bits were taken from the representative areas.

**Figure 2:**
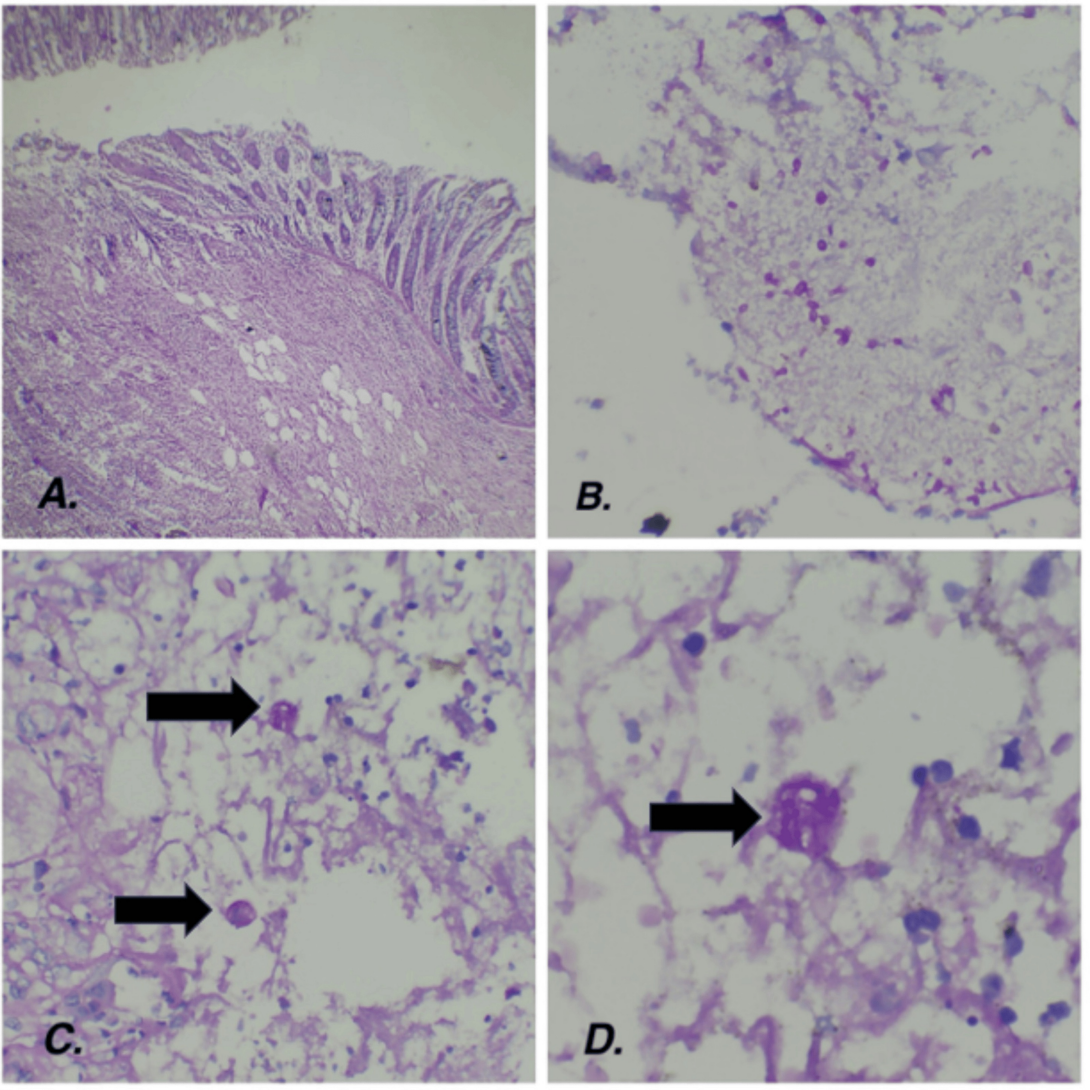
Histopathological examination 2A. Photomicrograph of the deep borrowing ulcers with areas of necrosis (Hematoxylin and eosin stain, 10x); 2B. Photomicrograph of the candida fungal spores (Peroxidase acid Schiff, 40x); 2C. (40x); 2D. (100x) Photomicrograph of the peroxidase acid Schiff positive trophozoites of *Entamoeba histolytica* with engulfed red blood cells intermixed in a background of inflammatory cell exudate

On microscopic examination, there were large areas of ulceration (Figure 2A), necrosis, transmural inflammation, perforation, dense serosal inflammation, and extensive fibrosis. The blood vessels showed marked thickening, fresh thrombi, and fibrinoid necrosis. Periodic acid shift staining was done, which showed organisms morphologically resembling entamoeba with ingested red blood cells in the cytoplasm, and there was superadded colonization of fungal organisms resembling Candida. (Figures 2B-2D). The regional lymph nodes were examined and showed reactive changes. The appendix was grossly and microscopically normal. The final diagnosis was given as colonic perforation at the splenic flexure with organisms conforming to the morphology of *Entamoeba histolytica* and superadded Candida colonization.

## Discussion

*Entamoeba histolytica*, an anaerobic parasitic amoebozoan, is endemic in many developing regions and has a higher prevalence in areas with poor sanitation and overcrowding. Estimates suggest that approximately 50 million people worldwide are infected, with a significant proportion experiencing symptomatic disease and about 100,000 deaths yearly [2]. Colonic perforation due to amoebic colitis is rare but serious and can occur in a small subset of patients with severe amoebic dysentery or those who have delayed treatment.

*Entamoeba histolytica* can penetrate the colonic mucosa by releasing proteolytic enzymes and various other factors that enhance its virulence. The organism can adhere to the intestinal wall and invade and disrupt the mucosal barrier causing contact-dependent killing and stimulating apoptosis of the intestinal cells [3]. This invasion leads to inflammation and ulceration, and in severe cases, can lead to tissue necrosis. Colonic perforation occurs when the pathological process weakens the colonic wall, resulting in a breach that allows fecal matter to enter the peritoneal cavity, which can lead to peritonitis and systemic infection. Factors that contribute to perforation include the extent of mucosal damage, the host's immune response, and the presence of other predisposing conditions such as malnutrition or concurrent infections. Studies suggest that the incidence of colonic perforation in patients with amoebic colitis is relatively low compared to the total number of amoebic colitis cases. It is estimated that perforation occurs in less than 1% of patients with amoebic colitis [4]. Tomino et al. present a case of fulminant amoebic colitis causing lethal colonic necrosis and perforation [5]. Ozdogan et al. state that amoebiasis causing perforation is a rare form of amoebiasis associated with high morbidity and mortality [6].

Candida infections are usually opportunistic and complicate conditions in immunocompromised or critically ill patients. The prevalence of Candida superinfection in amoebic colitis is not well-documented but is likely higher in patients with extensive disease or those receiving broad-spectrum antibiotics. Davis et al. describe a case of massive perianal ulceration with numerous amoebic trophozoites and periodic acid Schiff (PAS)-positive spores and pseudohyphal structures indicating yeast superinfection [7].

Peng et al. report a case of colonic amoebic abscess and state how, initially, amoeboma is often misdiagnosed as other surgically related intestinal diseases like carcinoma, diverticulitis, and Chron's disease, especially in the elderly [8].

## Conclusions

Amoebic colitis presenting as a colonic perforation is a rare complication that needs early diagnosis and intervention. The addition of Candida fungal infection further complicates the clinical scenario and requires a multidisciplinary approach to address both the primary amoebic infection and the secondary fungal overgrowth. Understanding the pathophysiology and the interplay between these infections is crucial for effective management.
